# Collapsing-based and kernel-based single-gene analyses applied to Genetic Analysis Workshop 17 mini-exome data

**DOI:** 10.1186/1753-6561-5-S9-S117

**Published:** 2011-11-29

**Authors:** Lun Li, Wei Zheng, Joon Sang Lee, Xianghua Zhang, John Ferguson, Xiting Yan, Hongyu Zhao

**Affiliations:** 1 Division of Biostatistics, Yale School of Public Health, Yale University, 60 College St., PO Box 208034, New Haven, CT 06520-8034, USA; 2 Hubei Bioinformatics and Molecular Imaging Key Laboratory, Huazhong University of Science and Technology, Wuhan, Hubei 430074, China; 3 Keck Biotechnology Resource Laboratory, Yale University, 300 George St., New Haven, CT 06511, USA; 4 Department of Electronic Science and Technology, University of Science and Technology of China, Hefei, Anhui 230027, China

## Abstract

Recently there has been great interest in identifying rare variants associated with common diseases. We apply several collapsing-based and kernel-based single-gene association tests to Genetic Analysis Workshop 17 (GAW17) rare variant association data with unrelated individuals without knowledge of the simulation model. We also implement modified versions of these methods using additional information, such as minor allele frequency (MAF) and functional annotation. For each of four given traits provided in GAW17, we use the Bayesian mixed-effects model to estimate the phenotypic variance explained by the given environmental and genotypic data and to infer an individual-specific genetic effect to use directly in single-gene association tests. After obtaining information on the GAW17 simulation model, we compare the performance of all methods and examine the top genes identified by those methods. We find that collapsing-based methods with weights based on MAFs are sensitive to the “lower MAF, larger effect size” assumption, whereas kernel-based methods are more robust when this assumption is violated. In addition, many false-positive genes identified by multiple methods often contain variants with exactly the same genotype distribution as the causal variants used in the simulation model. When the sample size is much smaller than the number of rare variants, it is more likely that causal and noncausal variants will share the same or similar genotype distribution. This likely contributes to the low power and large number of false-positive results of all methods in detecting causal variants associated with disease in the GAW17 data set.

## Background

To date, genome-wide association studies (GWAS) have been successful in unveiling many common single-nucleotide polymorphisms (SNPs) associated with common diseases, including type 1 and type 2 diabetes, rheumatoid arthritis, Crohn’s disease, and coronary heart disease [1-3]. However, the results from recent GWAS account for a relatively small proportion of the heritability of those diseases. One possible explanation of this limitation is that GWAS have focused mainly on variants that are common (minor allele frequency [MAF] > 5%), whereas many disease-causing variants may be rare and therefore difficult to tag using common variants.

The advent of next-generation sequencing technology has offered great opportunities for discovering novel rare variants in the human genome, associating these rare variants with diseases, and increasing our biological knowledge of disease etiology. In particular, as pointed out by Choi et al. [4], protein-coding regions harbor 85% of the mutations with large effects on disease-associated traits. As a result, whole-exome sequencing technology has emerged as a powerful paradigm for the identification of rare variants associated with diseases. This technology was used in the pilot3 study of the 1000 Genomes Project [5], from which the Genetic Analysis Workshop 17 (GAW17) mini-exome data were generated.

In the GAW17 mini-exome data set [6], most of the SNPs are rare (MAF < 5% for 21,355 out of 24,487 SNPs) so that multimarker association tests are more desirable than single-marker tests, such as the chi-square test, because of the potential to increase power from multiple signals in a region. However, because of higher degrees of freedom, multimarker association tests may have reduced power. To overcome this problem, investigators have recently proposed several multimarker association tests for which the test statistics have smaller degrees of freedom. In this paper, we consider two types of such association test procedures. The first approach is based on collapsing multimarkers within a chromosomal region to generate a reduced set of genetic predictors [7-9]; the second approach correlates genetic similarity among individuals across a set of markers by using a kernel function with their phenotypic similarity [10-13]. We describe these methods in the Methods section.

We apply these methods to each of the genes in the GAW17 unrelated individuals data set to identify genes associated with the given traits (Affected, Q1, Q2, and Q4), adjusting for the effects of environmental covariates (Smoke, Age, Sex, and Population). The results from these methods are compared. In addition, for each given trait, we use the Bayesian mixed-effects model to estimate the phenotypic variance that can be explained by the given environmental and genotypic data and to infer an individual-specific genetic effect to use directly in single-gene association tests.

## Methods

Let *X_i_* denote the vector of given environmental covariates such as Age and Sex, and let *Y_i_* denote the vector of a quantitative or qualitative trait for individual *i* (*i* = 1, 2, …, 697). Our general framework can be described as follows. For a binary trait,(1)

and for a quantitative trait,(2)

where *G_ik_* is a vector of minor allele counts for SNPs within gene *k* for individual *i*. In this framework, *h*(·) represents the genetic effect, adjusting for the effects of covariates *X_i_*. Then our main focus is on hypothesis testing for *h*(·) = 0 for each gene *k*.

### Collapsing-based methods

The collapsing method was first introduced by Li and Leal [7] for detecting disease associations. In this method, rare variants (MAF < 0.05) in gene *k* are collapsed so that one genetic variable *g_ik_* is obtained from *G_ik_* using an indicator function for the presence of rare variants in this gene for each individual *i*. Morris and Zeggini [8] extended this idea into a linear regression framework for quantitative traits and also introduced an alternative genetic variable *g_ik_*, based on *G_ik_*, defined by the proportion of rare variants. In a groupwise association test procedure proposed by Madsen and Browning [9] a new genetic variable *g_ik_* is defined through a weighted sum of the mutation counts based on their MAFs. As shown in Eqs. (1) and (2), we would like to take into account environmental covariates in our testing models; these covariates are not included in the testing procedures just described [7,9]. Therefore we borrow all the coding schemes of *g_ik_* for each *G_ik_* and model *h*(*G_ik_*) as *h*(*G_ik_*) = *β* · *g_ik_*. Then association testing is reduced to testing for *β* = 0.

As suggested by Li and Leal [7], markers can be divided into subgroups on the basis of predefined criteria. In this analysis, by using functional annotation information, we divide variants into synonymous and nonsynonymous groups. In this grouping scheme, ambiguously annotated SNPs (labeled unknown or empty) are combined with synonymous SNPs. By using the weighted sum of the mutation counts, we obtain genetic scores for nonsynonymous and synonymous groups and apply the models in Eqs. (1) and (2) to those two scores, that is,(3)

Then we perform association testing for *β_ns_* = *β_syn_* = 0.

### Kernel-based methods

An alternative powerful multimarker association test is the kernel-based association test (KBAT) [10,11]. KBATs are based on flexible high-dimensional data analysis techniques called the least-squares kernel machine (LSKM) for quantitative traits and the logistic kernel machine (LKM) for binary traits. Liu et al. [12,13] proposed the LSKM (LKM) method to relate continuous (binary) outcomes with covariates and the pathway effect of multiple gene expressions. For quantitative traits, *β* and *h* are estimated by maximizing the penalized likelihood function:(4)

where *λ* is a tuning parameter. The representer theorem by Kimeldorf and Wahba [14] shows that the solution to the nonparametric function *h*(·) can be expressed as:(5)

for a given kernel function *k*(·, ·). Then the estimates of *β* and *α* (equivalently, *h*) can be easily obtained by plugging the *h*(*G*) obtained from Eq. (5) into the penalized likelihood function (Eq. (4)). For more details on the estimation, see Wu et al. [11]. The relationship between the LSKM and linear mixed models leads to the assumption that *h*(·) ~ *N*(0, *τK*), where *τ* is a scalar and *K* is an *n* × *n* matrix whose (*i*, *j*)th component is *K*(*G_ik_*, *G_jk_*). As a result, testing hypothesis *h* = 0 is simply reduced to testing *τ* = 0. For the hypothesis testing for *τ* = 0, a score test statistic proposed by Zhang and Lin [15] can be used. This method has also been extended to case-control data by using the LKM approach [11]. KBAT methods are just the extension of the LSKM and LKM for multimarker associations.

Note that a prespecified kernel function *K*(*G_ik_*, *G_jk_*) measures the genetic similarity between two individuals *i* and *j* on the basis of their genotypes at the SNPs in gene *k*. If:(6)

then  implies that the genetic similarity to individual *j* does not influence  and thus estimates trait . In this analysis, we use a kernel function based on the number of alleles shared identical by state (IBS) by two individuals *i* and *j* at the SNPs within gene *k*. If *G_ik_* = (*M*_1_*_ik_*, …, *M_sik_*), where *M_rik_* denotes the genotype of individual *i* at SNP *r* in gene *k*, then a weighted IBS kernel can be defined by:(7)

where *w_lk_* is a weight based on *q_lk_*, the MAF of SNP *l* within gene *k*, and is defined by:(8)

here. For an unweighted IBS kernel, *w_lk_* is replaced by a constant, say, 1. The underlying idea behind the weighted IBS kernel is that similarity in rare alleles is more informative than similarity in common alleles for the trait similarity between two individuals so that the IBS kernel weights similarity in rarer alleles more.

### Bayesian mixed-effects model to estimate genetic effects of traits

We propose a Bayesian mixed-effects model to jointly analyze 200 simulation replicates. The main idea of our Bayesian mixed-effects model is to treat the genetic effect for each individual as a random effect and the environmental effect as a fixed effect. For disease status, we consider the logistic regression framework:(9)

and use the linear regression framework for Q1, Q2, and Q4, that is,(10)

where *e_ik_* ~ *N*(0, *σ*^2^), *k* = 1, …, 200, is the index for replicates and *i* = 1, …, 697 is the index for individuals. In both models, *g_i_* is the genetic effect of individual *i* and  is the environmental effect. To complete the Bayesian model, we specify the prior distribution for the model parameters as follows: *g_i_* ~ *N*(0, ) and *β_E_* ~ *N*(0, *Σ_β_*), in which *Σ_β_* is a diagonal matrix. The diagonal elements of *Σ_β_*, , and *σ*^2^ are further assigned noninformative inverse gamma distributions. For each trait, we fit the model using the Markov chain Monte Carlo algorithm.

## Results

### Variance of different traits explained by genetic effects

During the first round of association tests for different traits, we noticed a dramatic difference in the number and magnitude of significantly associated genes and environmental variables. Therefore we suspect that the variance in different traits that can be explained by the provided genotype data and environmental components may vary.

To estimate the upper limit of the explainable proportion of variance, we proposed a Bayesian mixed-effects model and compared the posterior means of *Σ_β_*, , and *σ*^2^. We found that Q1 is affected by both given genotype data and environmental variables; in contrast, Q2 is mainly affected by genetic but not environmental variables, and Q4 is not affected by any given genotypic data (Table 1).

**Table 1:** Proportion of phenotypic variance explained by environmental variables and genotypic data

Trait	Variance explained by genotypic data	Variance explained by environmental variables	Residual variance
Q1	0.206	0.161	0.633
Q2	0.124	0.008	0.868
Q4	0	0.787	0.213

Although this procedure was performed without knowing the GAW17 simulation answers, the observed pattern agrees well with the answers. Because Q4 is obviously not affected by any genotypes, we did not consider Q4 further in gene-level association tests.

### Investigation of top genes associated with disease status from different methods

Using the genetic effects *g_i_* estimated from the Bayesian mixed-effects model as responses, we applied three well-established collapsing methods and a kernel-based method to the GAW17 data set of 697 unrelated individuals and conducted gene-based association tests. To incorporate functional annotation information, we also separated nonsynonymous from synonymous SNPs in all methods and applied the modified versions too.

Table 2 lists the top 10 genes associated with disease identified by the different methods. The true causal gene *PIK3C3* was identified by all methods, probably because of its relatively large effect size and MAF. The true causal gene *PIK2B* was identified by methods considering both synonymous and nonsynonymous SNPs but dropped off the top 10 list for methods considering only nonsynonymous SNPs. Interestingly, the combined multivariate and collapsing (CMC) synonymous method, which examined only noise variables, also reported *PIK2B* in the top 10 gene list, indicating that some synonymous variants in *PIK2B* also contain association signals. Indeed, we found that a noncausal synonymous SNP (C8S886) in *PIK2B* had an identical genotype distribution with a causal SNP (C5S5156) in *FLT4* (a causal gene for Q1 that indirectly affected disease status).

**Table 2:** Top 10 disease-associated genes from different methods

Collapsing	Weighted-sum	Kernel (weighted IBS)	CMC (both synonymous and nonsynonymous SNPs)	CMC (synonymous SNPs only)	Collapsing (nonsynonymous SNPs only)	Weighted sum (nonsynonymous SNPs only)	Kernel (weighted IBS, nonsynonymous SNPs only)	CMC (nonsynonymous SNPs only)	Kernel (IBS, nonsynonymous SNPs only)
***PIK3C3***	***PIK3C3***	**FLT1**	**FLT1**	PRH1	**FLT1**	**FLT1**	MAP3K6	**FLT1**	OR2T3
**FLT1**	**FLT1**	TAS2R48	PRH1	TAS2R48	***PIK3C3***	***PIK3C3***	NOTCH2NL	***PIK3C3***	OR2T34
PRH1	PRH1	PRH1	***PIK3C3***	ZNF91	**KDR**	**KDR**	**FLT1**	**KDR**	HLA-A
PRR4	PRR4	PRR4	OR52E4	***PTK2B***	KCNJ12	OR52E4	OR2T34	OR2T3	OR52E4
***PTK2B***	***PTK2B***	***PIK3C3***	TAS2R48	LOC645118	ZNF77	NOTCH2NL	RGPD4	MAP3K6	**FLT1**
ZNF91	ZNF91	SUSD2	**KDR**	INSR	NOTCH2NL	ZNF77	LRP1B	HLA-L	KCNJ12
NOTCH2NL	NOTCH2NL	KCNJ12	NOTCH2NL	TERT	OR9G1	BRCA1	***PIK3C3***	**VNN1**	***PIK3C3***
TAS2R48	TAS2R48	OR52E4	***PTK2B***	EPHB1	OR2T3	OR9G1	**VNN1**	PATE	**SUSD2**
MUSK	MUSK	HLA-B	ZNF91	PRR4	EPHA5	OR2T3	MYO3A	E2F2	HLA-L
KIT	KIT	***PTK2B***	LRP1B	TNK1	E2F2	MAP3K6	TACC2	C1ORF147	SSTR4

For the kernel-based methods, there are 43 genes (Kernel, weighted IBS) and 19 genes (Kernel, weighted IBS, nonsynonymous SNPs only) that have *p*-values less than 10^−16^ and thus are reported as 0. These genes cannot be ranked effectively. Genes in bold and italic are causal genes for disease liability; genes in bold are associated with Q1 or Q2.

Some false-positive genes were often identified by multiple methods for similar reasons. For example, the false-positive gene *NOTCH2NL* contains a SNP (C1S6297) that is identical with C18S2475 in *PIK3C3*. The false-positive genes *PRH1*, *PRR4*, and *TAS2R48* are colocated on chromosome 12 and share SNP C12S717, which has the same genotype distribution as C7S5144, a causal variant for Q2. The false-positive gene *SUSD2* contains the SNP C22S929, which is identical with causal variants C1S3181 in *ELAVL4* (associated with Q2) and C6S5448 in *VNN3* (associated with disease status). The false-positive gene *KIT* contains C4S1839, which is close to and identical with the causal variant C4S1873 in *KDR*. Some other commonly identified false-positive genes (e.g., *MUSK* and *ZNF91*) share similar but not exactly the same genotype distributions with causal genes (e.g., *PRKCA* and *PTK2B*), and their genetic scores are highly correlated (*p* < 2.2 × 10^−16^).

In summary, there are many confounded signals in the GAW17 data set. We found 1,494 SNPs sharing exactly the same genotype distribution with at least one of the 160 causal SNPs. This posed a big challenge in the identification of causal genes, especially for traits with a large number of underlying causal variants, such as disease status. This may be a common problem in rare variant association studies because the sample size is usually much smaller than the number of variants. When most variants have extremely low MAFs, it is likely that their genotype distributions will coincide.

### Comparison of collapsing- and kernel-based methods

**Figure 1:**
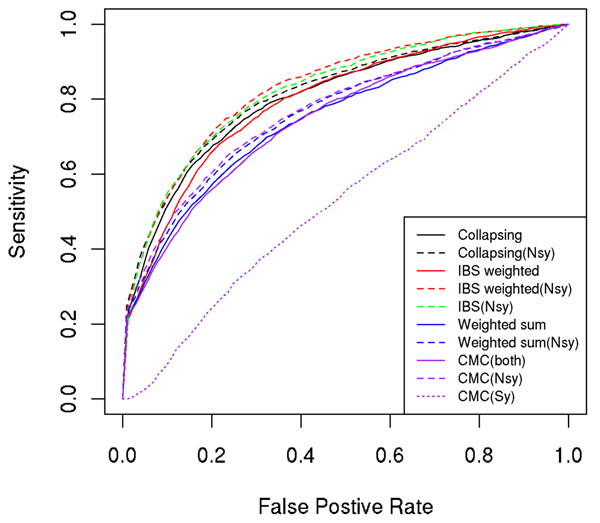
ROC curves for Q1

**Figure 2:**
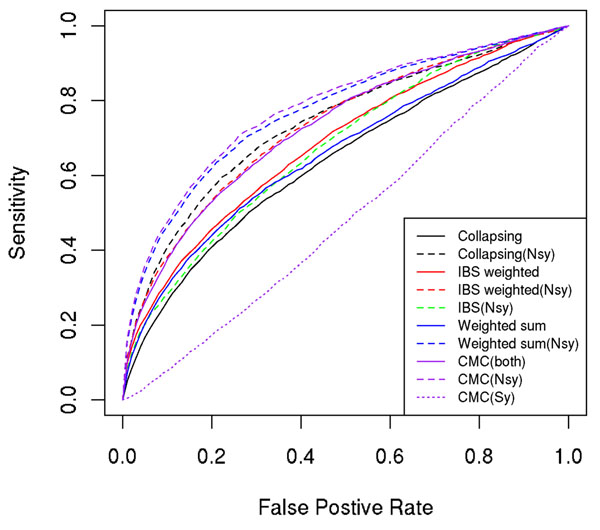
ROC curves for Q2

**Figure 3:**
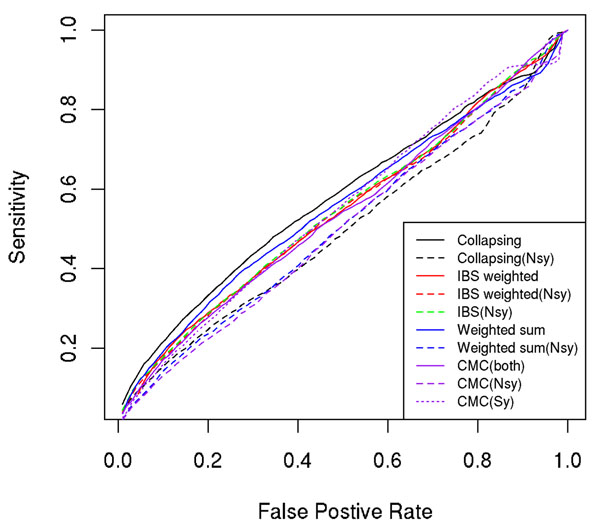
ROC curves for disease status

After obtaining the simulation answers from the GAW17 meetings, we analyzed 200 simulated data sets and then counted how many causal genes and false-positive genes were identified by each method at different significance thresholds, and we plotted receiver operating characteristic (ROC) curves for all methods (Figures 1, 2, 3). From the plots, we found that all methods lacked power to identify disease causal genes (Figure 1). However, these methods were able to identify some true signals for Q1 (Figure 2) and Q2 (Figure 3). Methods considering only nonsynonymous variants (dashed lines in the ROC plots) performed consistently better than their counterparts using both nonsynonymous and synonymous variants; this was expected because the simulation model involved only nonsynonymous SNPs. This is probably true for real data as well because nonsynonymous SNPs are more likely to change protein structure and to have larger biological effects.

Another pattern revealed by the ROC plots for Q1 and Q2 is that the weighted-sum and CMC methods that assign more weight to rarer variants performed worse than other methods for Q1 and comparable to other methods for Q2. This is probably because the “lower MAF, larger effect” assumption does not hold for Q1. We checked the correlation between MAF and effect size (*β*) of causal variants for Q1 and Q2 and found that correlation for Q1 (−0.17) is not significantly different from 0 (*p* = 0.3), whereas correlation for Q2 (−0.23) is only marginally significant (*p* = 0.05). Interestingly, the kernel method using a weighted IBS kernel did not suffer much power loss in Q1, although it also assigned more weight to rarer variants. It performed favorably and at least as good as two baseline methods (collapsing and weighted-sum) no matter whether the assumption was true or not. In real data, when we do not know whether rarer variants have larger effect sizes, the kernel-based method is preferable.

## Discussion

A key contribution of our work is the application of the kernel-based method in the setting of association tests with rare variants. Originally this method was proposed in common variant association studies to enrich signals from multiple genotypic markers and to reduce the degrees of freedom in association tests. We found it suitable for rare variant association studies as well because single-marker tests using rare variants have low power as a result of the extremely low MAFs. To our knowledge, the kernel-based method has not been widely applied to rare variant association studies, and our systematic comparisons of this method with other well-established collapsing methods provide a better understanding of its behavior and potential use in rare variant association studies.

Another novel contribution we make is the application of a Bayesian mixed-effects model. This procedure makes use of all 200 simulation replicates and serves two purposes. First, by comparing the posterior mean of *Σ_β_*, , and *σ*^2^, we can estimate the proportion of phenotypic variation that can be explained by environmental variables and given genotype data. Second, the posterior mean of *g_i_* is treated as a new response without environmental covariate effects and is directly used in association tests with genotypic data. It provides the basis for a more reliable comparison of different collapsing-based and kernel-based association methods by evaluating the result consistency across different replicates.

## Conclusions

We have two major conclusions. First, collapsing-based methods that assign more weight to rarer variants are sensitive to the “lower MAF, larger effect size” assumption, whereas kernel-based methods are more robust and suffer less power loss, even when the assumption is violated. Second, many false-positive genes identified by multiple methods often contain SNPs with exactly the same genotype distribution as the causal variants used in the simulation model. When sample size is much smaller than the number of rare variants, it is likely that the causal and noncausal variants will share the same or similar genotype distributions. This might lead to poor power and a large number of false-positive results for all methods in detecting causal disease-associated variants in the GAW17 data set.

## Competing interests

The authors declare that there are no competing interests.

## Authors’ contributions

All authors participated in the design of the study. LL, WZ and JL performed statistical analysis and drafted the manuscript. JF and XZ proposed and implemented the Bayesian mixed-effects model. XY made valuable suggestions and helped to organize the study. HZ coordinated and helped to draft the manuscript. All authors read and approved the final manuscript.
